# Aqua­bis(2-iodo­acetato-κ*O*)(1,10-phenanthroline-κ^2^ *N*,*N*′)copper(II)

**DOI:** 10.1107/S1600536809002682

**Published:** 2009-01-31

**Authors:** Rengao Zhao, Junshan Sun, Jie Lu, Jikun Li

**Affiliations:** a Department of Materials and Chemical Engineering, Taishan University, 271021 Taian, Shandong, People’s Republic of China; b Department of Applied and Science Technology, Taishan University, 271021 Taian, Shandong, People’s Republic of China

## Abstract

In the title compound, [Cu(C_2_H_2_IO_2_)_2_(C_12_H_8_N_2_)(H_2_O)], the Cu^II^ ion is coordinated by two N atoms [Cu—N = 2.013 (4) and 2.024 (4) Å] from a 1,10-phenanthroline ligand and three O atoms [Cu—O = 1.940 (4)–2.261 (4) Å] from two carboxyl ligands and a water mol­ecule in a distorted square-pyramidal geometry. One iodo­acetate O atom [Cu—O = 2.775 (4) Å] completes the coordination to form a distorted octa­hedron. Inter­molecular O—H⋯O hydrogen bonds link the mol­ecules into centrosymmetric dimers, which are further packed by π–π inter­actions between the 1,10-phenanthroline ligands into layers parallel to the *ab* plane. The crystal packing also exhibits short inter­molecular I⋯I contacts of 3.6772 (9) Å and weak C—H⋯O hydrogen bonds.

## Related literature

The related crystal structure of aqua­bis(2,4-dichlorophenoxy­acetato-*O*)(1,10-phenanthroline-κ^2^ *N*,*N*′)copper(II) has been reported by Liu *et al.* (2006 ▶).
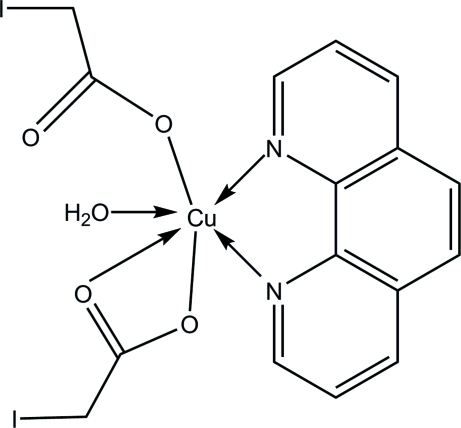


## Experimental

### 

#### Crystal data

[Cu(C_2_H_2_IO_2_)_2_(C_12_H_8_N_2_)(H_2_O)]
*M* *_r_* = 631.63
Triclinic, 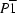

*a* = 9.5156 (11) Å
*b* = 10.6293 (12) Å
*c* = 11.3441 (13) Å
α = 65.803 (2)°
β = 65.598 (2)°
γ = 72.451 (2)°
*V* = 940.94 (19) Å^3^
*Z* = 2
Mo *K*α radiation
μ = 4.47 mm^−1^
*T* = 273 (2) K
0.26 × 0.23 × 0.21 mm

#### Data collection

Bruker SMART APEX diffractometer
Absorption correction: multi-scan (*SADABS*; Sheldrick, 1996 ▶) *T*_min_ = 0.389, *T*_max_ = 0.454 (expected range = 0.336–0.391)
4948 measured reflections
3305 independent reflections
2934 reflections with *I* > 2σ(*I*)
*R*_int_ = 0.016

#### Refinement

*R*[*F* ^2^ > 2σ(*F* ^2^)] = 0.038
*wR*(*F* ^2^) = 0.104
*S* = 1.01
3305 reflections
237 parameters
3 restraints
H-atom parameters constrained
Δρ_max_ = 1.53 e Å^−3^
Δρ_min_ = −1.68 e Å^−3^

### 

Data collection: *SMART* (Siemens, 1996 ▶); cell refinement: *SAINT* (Siemens, 1996 ▶); data reduction: *SAINT*; program(s) used to solve structure: *SHELXS97* (Sheldrick, 2008 ▶); program(s) used to refine structure: *SHELXL97* (Sheldrick, 2008 ▶); molecular graphics: *SHELXTL* (Sheldrick, 2008 ▶); software used to prepare material for publication: *SHELXTL* (Sheldrick, 2008 ▶).

## Supplementary Material

Crystal structure: contains datablocks I, global. DOI: 10.1107/S1600536809002682/cv2511sup1.cif

Structure factors: contains datablocks I. DOI: 10.1107/S1600536809002682/cv2511Isup2.hkl

Additional supplementary materials:  crystallographic information; 3D view; checkCIF report

## Figures and Tables

**Table 1:** Selected interatomic distances (Å) *Cg*1, *Cg*2 and *Cg*3 are the centroids of the C4–C7/C11/C12, C6–C10/N2 and C1–C5/N1 rings, respectively.

*Cg*1⋯*Cg*3^i^	3.505 (6)
*Cg*1⋯*Cg*1^ii^	3.584 (6)
*Cg*2⋯*Cg*3^i^	3.625 (6)
*Cg*2⋯*Cg*1^ii^	3.634 (6)
I2⋯I2^iii^	3.6772 (9)

Symmetry codes: (i) 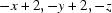; (ii) 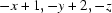; (iii) 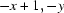, 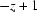.

**Table 2:** Hydrogen-bond geometry (Å, °)

*D*—H⋯*A*	*D*—H	H⋯*A*	*D*⋯*A*	*D*—H⋯*A*
O1—H1*B*⋯O3	0.85	1.84	2.639 (6)	156
O1—H1*C*⋯O4^iv^	0.85	1.97	2.785 (5)	161
C3—H3⋯O1^ii^	0.93	2.44	3.240 (7)	144
C11—H11⋯O5^i^	0.93	2.71	3.508 (8)	144
C10—H10⋯O3^iv^	0.93	2.68	3.431 (8)	138
C14—H14*B*⋯O2^v^	0.97	2.59	3.436 (8)	146
C14—H14*A*⋯O5^v^	0.97	2.64	3.219 (8)	119

Symmetry codes: (i) 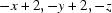; (ii) 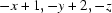; (iv) 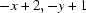, 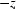; (v) 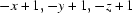.

**Fig. 1.**
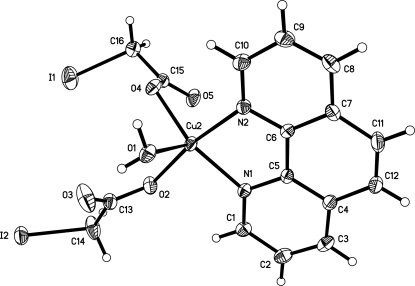
The molecular structure of the title compound showing the atomic numbering and 30% probability displacement ellipsoids.

## supplementary crystallographic information

### Comment

Metal complexes with carboxylates are among the most investigated complexes in the field of coordination chemistry. Due to their versatile bonding modes with metal ions, they have also been used in the synthesis of mononuclear monomeric and polymeric complexes (Liu *et al.*, 2006). In order to develop some new topological structures, we study the reaction of the copper(II) ion and 2-iodoacetic acid with the presence of 1,10-phenanthroline.

The molecular structure of the title complex is shown in Fig.1. The Cu atom exhibits a six-coordinated distorted octahedral pyramidal geometry with two carboxyl O atoms from (Cu2—O4 2.000 (4) Å, Cu2—O5 2.775 (4) Å), a water molecule (Cu—O 2.261 (4) Å) and a nitrogen atom (Cu2—N2 2.024 (4) Å) occupying the equatorial planar position. A nitrogen atom N2 (Cu2—N2 2.013 (4) Å) and a carboxyl O atom (Cu2—O2 1.940 (4) Å) occupy the apical positions. The displacement of the metal atom from the basal plane is 0.0640 (2) Å. The crystal packing exhibits short intermolecular I···I contacts (Table 1) and weak C—H···O hydrogen bonds (Table 2).

### Experimental

The reaction was carried out by the solvothermal method. 2-iodoacetic acid(0.372 g,2 mmol) and cupric acetate(0.199 g, 1 mmol) and 1,10-phenanthroline(0.180 g, 1 mmol) were added to the airtight vessel with 20 ml water. The resulting green solution was filtered. The filtrate was placed for sevaral days yielding blue block-shaped crystals.

The yield is 81%. Elemental analysis: calc. for C_16_H_14_CuI_2_N_2_O_5_: C 30.42, H 2.23, N 4.43; found: C 30.15, H 2.49, N 4.22. The elemental analyses were performed with PERKIN ELMER MODEL 2400 SERIES II.

### Refinement

All the H atoms were found in Fourier map, but placed in idealized positions(C—H 0.93–0.97 Å, O—H 0.85 Å), with the *U*_iso_(H) values were set at 1.2*U*_eq_(C,*O*) of the parent atoms.

### Crystal data

**Table:**

[Cu(C_2_H_2_IO_2_)_2_(C_12_H_8_N_2_)(H_2_O)]	*Z* = 2
*M**_r_* = 631.63	*F*(000) = 598
Triclinic, *P*1	*D*_x_ = 2.229 Mg m^−^^3^
*a* = 9.5156 (11) Å	Mo *K*α radiation, λ = 0.71073 Å
*b* = 10.6293 (12) Å	Cell parameters from 3047 reflections
*c* = 11.3441 (13) Å	θ = 2.6–28.1°
α = 65.803 (2)°	µ = 4.47 mm^−^^1^
β = 65.598 (2)°	*T* = 273 K
γ = 72.451 (2)°	Block, blue
*V* = 940.94 (19) Å^3^	0.26 × 0.23 × 0.21 mm

### Data collection

**Table:**

Bruker APEXII diffractometer	3305 independent reflections
Radiation source: fine-focus sealed tube	2934 reflections with *I* > 2σ(*I*)
graphite	*R*_int_ = 0.016
φ and ω scans	θ_max_ = 25.1°, θ_min_ = 2.1°
Absorption correction: multi-scan (*SADABS*; Sheldrick, 1996)	*h* = −11→11
*T*_min_ = 0.389, *T*_max_ = 0.454	*k* = −12→10
4948 measured reflections	*l* = −13→12

### Refinement

**Table:**

Refinement on *F*^2^	Primary atom site location: structure-invariant direct methods
Least-squares matrix: full	Secondary atom site location: difference Fourier map
*R*[*F*^2^ > 2σ(*F*^2^)] = 0.038	Hydrogen site location: inferred from neighbouring sites
*wR*(*F*^2^) = 0.104	H-atom parameters constrained
*S* = 1.01	*w* = 1/[σ^2^(*F*_o_^2^) + (0.056*P*)^2^ + 3.6149*P*] where *P* = (*F*_o_^2^ + 2*F*_c_^2^)/3
3305 reflections	(Δ/σ)_max_ < 0.001
237 parameters	Δρ_max_ = 1.53 e Å^−^^3^
3 restraints	Δρ_min_ = −1.68 e Å^−^^3^

### Special details

**Table:**

Geometry. All e.s.d.'s (except the e.s.d. in the dihedral angle between two l.s. planes) are estimated using the full covariance matrix. The cell e.s.d.'s are taken into account individually in the estimation of e.s.d.'s in distances, angles and torsion angles; correlations between e.s.d.'s in cell parameters are only used when they are defined by crystal symmetry. An approximate (isotropic) treatment of cell e.s.d.'s is used for estimating e.s.d.'s involving l.s. planes.
Refinement. Refinement of *F*^2^ against ALL reflections. The weighted *R*-factor *wR* and goodness of fit *S* are based on *F*^2^, conventional *R*-factors *R* are based on *F*, with *F* set to zero for negative *F*^2^. The threshold expression of *F*^2^ > σ(*F*^2^) is used only for calculating *R*-factors(gt) *etc*. and is not relevant to the choice of reflections for refinement. *R*-factors based on *F*^2^ are statistically about twice as large as those based on *F*, and *R*- factors based on ALL data will be even larger.

### Fractional atomic coordinates and isotropic or equivalent isotropic displacement parameters (Å^2^)

**Table:**

	*x*	*y*	*z*	*U*_iso_*/*U*_eq_	
Cu2	0.76967 (7)	0.67482 (6)	0.13753 (6)	0.02670 (17)	
I1	0.95532 (7)	0.21497 (5)	0.43653 (6)	0.06644 (19)	
I2	0.43146 (5)	0.18948 (4)	0.45215 (4)	0.04345 (15)	
N1	0.6118 (5)	0.8490 (4)	0.1546 (4)	0.0255 (9)	
N2	0.9024 (5)	0.8189 (4)	−0.0095 (4)	0.0280 (9)	
O1	0.7777 (4)	0.6019 (4)	−0.0268 (4)	0.0341 (9)	
H1C	0.8711	0.5671	−0.0635	0.031 (15)*	
H1B	0.7272	0.5340	0.0231	0.06 (2)*	
O2	0.6142 (4)	0.5607 (4)	0.2794 (4)	0.0359 (9)	
O3	0.6083 (7)	0.4189 (6)	0.1825 (5)	0.0661 (15)	
O4	0.9474 (4)	0.5270 (4)	0.1793 (4)	0.0329 (8)	
O5	0.8852 (5)	0.6118 (4)	0.3468 (4)	0.0429 (10)	
C1	0.4672 (6)	0.8592 (6)	0.2411 (5)	0.0307 (11)	
H1A	0.4265	0.7782	0.3029	0.037*	
C2	0.3740 (7)	0.9891 (6)	0.2417 (6)	0.0382 (13)	
H2	0.2725	0.9934	0.3032	0.046*	
C3	0.4311 (7)	1.1090 (6)	0.1529 (6)	0.0382 (13)	
H3	0.3693	1.1954	0.1536	0.046*	
C4	0.5840 (6)	1.1012 (5)	0.0602 (6)	0.0302 (11)	
C5	0.6710 (6)	0.9677 (5)	0.0657 (5)	0.0241 (10)	
C6	0.8265 (6)	0.9512 (5)	−0.0244 (5)	0.0249 (10)	
C7	0.8936 (6)	1.0689 (6)	−0.1235 (5)	0.0305 (11)	
C8	1.0481 (7)	1.0437 (6)	−0.2087 (6)	0.0380 (13)	
H8	1.0990	1.1181	−0.2742	0.046*	
C9	1.1228 (7)	0.9109 (6)	−0.1952 (6)	0.0392 (13)	
H9	1.2240	0.8936	−0.2535	0.047*	
C10	1.0475 (6)	0.8002 (6)	−0.0934 (6)	0.0356 (12)	
H10	1.1012	0.7096	−0.0841	0.043*	
C11	0.8014 (8)	1.2041 (6)	−0.1279 (7)	0.0432 (14)	
H11	0.8440	1.2830	−0.1935	0.052*	
C12	0.6551 (7)	1.2204 (6)	−0.0396 (6)	0.0381 (13)	
H12	0.5997	1.3098	−0.0436	0.046*	
C13	0.5693 (6)	0.4601 (6)	0.2801 (6)	0.0336 (12)	
C14	0.4522 (9)	0.3957 (7)	0.4171 (7)	0.0550 (19)	
H14A	0.3507	0.4539	0.4228	0.066*	
H14B	0.4825	0.3950	0.4891	0.066*	
C15	0.9541 (6)	0.5230 (5)	0.2913 (5)	0.0293 (11)	
C16	1.0576 (7)	0.3988 (6)	0.3569 (6)	0.0354 (12)	
H16A	1.0700	0.4144	0.4303	0.043*	
H16B	1.1602	0.3872	0.2894	0.043*	

### Atomic displacement parameters (Å^2^)

**Table:**

	*U*^11^	*U*^22^	*U*^33^	*U*^12^	*U*^13^	*U*^23^
Cu2	0.0264 (3)	0.0205 (3)	0.0253 (3)	−0.0018 (2)	−0.0022 (3)	−0.0080 (2)
I1	0.0913 (4)	0.0355 (3)	0.0650 (3)	−0.0196 (2)	−0.0323 (3)	0.0035 (2)
I2	0.0464 (3)	0.0329 (2)	0.0460 (3)	−0.01190 (17)	−0.00496 (18)	−0.01484 (18)
N1	0.028 (2)	0.025 (2)	0.022 (2)	−0.0010 (17)	−0.0070 (17)	−0.0096 (17)
N2	0.029 (2)	0.026 (2)	0.026 (2)	−0.0039 (18)	−0.0058 (18)	−0.0100 (18)
O1	0.036 (2)	0.032 (2)	0.0267 (19)	0.0013 (18)	−0.0049 (16)	−0.0137 (17)
O2	0.040 (2)	0.031 (2)	0.031 (2)	−0.0118 (17)	0.0021 (17)	−0.0139 (16)
O3	0.093 (4)	0.070 (3)	0.038 (3)	−0.050 (3)	0.012 (2)	−0.030 (2)
O4	0.034 (2)	0.0286 (19)	0.0270 (19)	0.0040 (16)	−0.0075 (16)	−0.0095 (16)
O5	0.049 (2)	0.034 (2)	0.044 (2)	−0.0013 (19)	−0.0081 (19)	−0.0224 (19)
C1	0.028 (3)	0.035 (3)	0.024 (3)	−0.003 (2)	−0.003 (2)	−0.012 (2)
C2	0.031 (3)	0.046 (3)	0.032 (3)	0.003 (3)	−0.007 (2)	−0.018 (3)
C3	0.036 (3)	0.037 (3)	0.043 (3)	0.009 (2)	−0.019 (3)	−0.019 (3)
C4	0.034 (3)	0.027 (3)	0.037 (3)	−0.001 (2)	−0.019 (2)	−0.013 (2)
C5	0.031 (3)	0.022 (2)	0.025 (2)	−0.002 (2)	−0.014 (2)	−0.009 (2)
C6	0.025 (3)	0.025 (3)	0.025 (2)	0.000 (2)	−0.010 (2)	−0.010 (2)
C7	0.035 (3)	0.029 (3)	0.030 (3)	−0.011 (2)	−0.013 (2)	−0.006 (2)
C8	0.040 (3)	0.042 (3)	0.032 (3)	−0.020 (3)	−0.010 (2)	−0.005 (2)
C9	0.031 (3)	0.049 (4)	0.032 (3)	−0.010 (3)	−0.001 (2)	−0.016 (3)
C10	0.032 (3)	0.038 (3)	0.031 (3)	−0.002 (2)	−0.002 (2)	−0.017 (2)
C11	0.055 (4)	0.024 (3)	0.051 (4)	−0.013 (3)	−0.025 (3)	−0.001 (3)
C12	0.042 (3)	0.025 (3)	0.050 (4)	−0.002 (2)	−0.022 (3)	−0.010 (3)
C13	0.033 (3)	0.030 (3)	0.031 (3)	−0.010 (2)	−0.001 (2)	−0.010 (2)
C14	0.071 (5)	0.048 (4)	0.039 (4)	−0.034 (4)	0.013 (3)	−0.022 (3)
C15	0.029 (3)	0.024 (3)	0.028 (3)	−0.007 (2)	−0.002 (2)	−0.007 (2)
C16	0.040 (3)	0.034 (3)	0.033 (3)	−0.003 (2)	−0.015 (3)	−0.010 (2)

### Geometric parameters (Å, °)

**Table:**

Cu2—O2	1.940 (4)	C3—C4	1.402 (8)
Cu2—O4	2.000 (4)	C3—H3	0.9300
Cu2—O5	2.775 (4)	C4—C5	1.402 (7)
Cu2—N2	2.013 (4)	C4—C12	1.433 (8)
Cu2—N1	2.024 (4)	C5—C6	1.416 (7)
Cu2—O1	2.261 (4)	C6—C7	1.404 (7)
I1—C16	2.134 (6)	C7—C8	1.403 (8)
I2—I2^i^	3.6772 (9)	C7—C11	1.434 (8)
I2—C14	2.117 (6)	C8—C9	1.352 (8)
N1—C1	1.322 (6)	C8—H8	0.9300
N1—C5	1.357 (6)	C9—C10	1.394 (8)
N2—C10	1.325 (7)	C9—H9	0.9300
N2—C6	1.349 (6)	C10—H10	0.9300
O1—H1C	0.8500	C11—C12	1.348 (9)
O1—H1B	0.8500	C11—H11	0.9300
O2—C13	1.262 (7)	C12—H12	0.9300
O3—C13	1.230 (7)	C13—C14	1.511 (8)
O4—C15	1.282 (6)	C14—H14A	0.9700
O5—C15	1.221 (6)	C14—H14B	0.9700
C1—C2	1.399 (8)	C15—C16	1.510 (7)
C1—H1A	0.9300	C16—H16A	0.9700
C2—C3	1.359 (9)	C16—H16B	0.9700
C2—H2	0.9300		
			
Cg1···Cg3^ii^	3.505 (6)	Cg2···Cg4^iii^	3.634 (6)
Cg1···Cg4^iii^	3.584 (6)	I2···I2^i^	3.6772 (9)
Cg2···Cg3^ii^	3.625 (6)		
			
O2—Cu2—O4	92.78 (16)	C7—C6—C5	120.1 (4)
O2—Cu2—N2	170.83 (17)	C8—C7—C6	116.6 (5)
O4—Cu2—N2	96.04 (17)	C8—C7—C11	125.3 (5)
O2—Cu2—N1	89.71 (17)	C6—C7—C11	118.1 (5)
O4—Cu2—N1	153.55 (16)	C9—C8—C7	119.8 (5)
N2—Cu2—N1	81.29 (17)	C9—C8—H8	120.1
O2—Cu2—O1	93.26 (15)	C7—C8—H8	120.1
O4—Cu2—O1	92.60 (14)	C8—C9—C10	119.7 (5)
N2—Cu2—O1	88.81 (16)	C8—C9—H9	120.2
N1—Cu2—O1	113.56 (15)	C10—C9—H9	120.2
C1—N1—C5	118.9 (4)	N2—C10—C9	122.7 (5)
C1—N1—Cu2	128.7 (4)	N2—C10—H10	118.7
C5—N1—Cu2	112.3 (3)	C9—C10—H10	118.7
C10—N2—C6	117.8 (5)	C12—C11—C7	122.0 (5)
C10—N2—Cu2	129.0 (4)	C12—C11—H11	119.0
C6—N2—Cu2	113.1 (3)	C7—C11—H11	119.0
Cu2—O1—H1C	109.3	C11—C12—C4	120.6 (5)
Cu2—O1—H1B	99.7	C11—C12—H12	119.7
H1C—O1—H1B	106.6	C4—C12—H12	119.7
C13—O2—Cu2	130.1 (3)	O3—C13—O2	126.2 (5)
C15—O4—Cu2	108.2 (3)	O3—C13—C14	122.0 (5)
N1—C1—C2	121.5 (5)	O2—C13—C14	111.7 (5)
N1—C1—H1A	119.2	C13—C14—I2	113.9 (4)
C2—C1—H1A	119.2	C13—C14—H14A	108.8
C3—C2—C1	120.4 (5)	I2—C14—H14A	108.8
C3—C2—H2	119.8	C13—C14—H14B	108.8
C1—C2—H2	119.8	I2—C14—H14B	108.8
C2—C3—C4	119.3 (5)	H14A—C14—H14B	107.7
C2—C3—H3	120.4	O5—C15—O4	125.0 (5)
C4—C3—H3	120.4	O5—C15—C16	118.5 (5)
C5—C4—C3	117.3 (5)	O4—C15—C16	116.5 (4)
C5—C4—C12	118.6 (5)	C15—C16—I1	109.7 (4)
C3—C4—C12	124.1 (5)	C15—C16—H16A	109.7
N1—C5—C4	122.6 (5)	I1—C16—H16A	109.7
N1—C5—C6	116.8 (4)	C15—C16—H16B	109.7
C4—C5—C6	120.6 (5)	I1—C16—H16B	109.7
N2—C6—C7	123.4 (5)	H16A—C16—H16B	108.2
N2—C6—C5	116.5 (4)		

Symmetry codes: (i) −*x*+1, −*y*, −*z*+1; (ii) −*x*+2, −*y*+2, −*z*; (iii) −*x*+1, −*y*+2, −*z*.

### Hydrogen-bond geometry (Å, °)

**Table:**

*D*—H···*A*	*D*—H	H···*A*	*D*···*A*	*D*—H···*A*
O1—H1B···O3	0.85	1.84	2.639 (6)	156
O1—H1C···O4^iv^	0.85	1.97	2.785 (5)	161
C3—H3···O1^iii^	0.93	2.44	3.240 (7)	144
C11—H11···O5^ii^	0.93	2.71	3.508 (8)	144
C10—H10···O3^iv^	0.93	2.68	3.431 (8)	138
C14—H14B···O2^v^	0.97	2.59	3.436 (8)	146
C14—H14A···O5^v^	0.97	2.64	3.219 (8)	119

Symmetry codes: (iv) −*x*+2, −*y*+1, −*z*; (iii) −*x*+1, −*y*+2, −*z*; (ii) −*x*+2, −*y*+2, −*z*; (v) −*x*+1, −*y*+1, −*z*+1.
